# Whole-Genome Sequences of Ralstonia solanacearum Strains P816, P822, and P824, Emerging Pathogens of Blueberry in Florida

**DOI:** 10.1128/MRA.01316-18

**Published:** 2019-01-17

**Authors:** Ana Maria Bocsanczy, Andres S. Espindola, David J. Norman

**Affiliations:** a Mid-Florida Research and Education Center (MREC), Institute of Food and Agricultural Sciences (IFAS), Plant Pathology, University of Florida, Apopka, Florida, USA; b Department of Entomology and Plant Pathology, Oklahoma State University, Stillwater, Oklahoma, USA; University of Delaware

## Abstract

Ralstonia solanacearum is the causal agent of bacterial wilt in numerous species of plants. Here, we report the whole-genome sequence of three phylogenetically diverse R. solanacearum strains, P816, P822, and P824, reported for the first time as causal agents of an emerging blueberry disease in Florida.

## ANNOUNCEMENT

During 2016 and 2017, there was an outbreak of diseased blueberry plants in Florida. Bronzing of leaves, marginal leaf necrosis, and bacterial streaming were observed in affected plants. The causal agent was identified as Ralstonia solanacearum (1), constituting an emerging pathogen of blueberry in Florida. Most commonly, when a new host for a bacterial pathogen is identified, isolated populations are clonal. Surprisingly, in this case, three phylogenetically diverse populations were isolated from blueberry in multiple counties in Florida (1). Here, we report the whole-genome sequences of P816 (phylotype IIA, sequevar 38), P822 (phylotype IIA, sequevar 7), and P824 (phylotype I, sequevar 13) that are representative strains of each distinct population of R. solanacearum infecting blueberry plants in Florida. All isolates were obtained from symptomatic blueberry plants as described previously (1). R. solanacearum strains picked from a single colony on a plate were grown at 28°C overnight in liquid Casamino Acid-peptone-glucose (CPG) medium (2). Genomic DNA was obtained using an Ultraclean microbial DNA isolation kit (catalog number, 12224-50; Mo Bio, CA) following the manufacturer’s instructions. Macrogen Corp. (Rockville, MD) performed the library preparation and Illumina sequencing. An Illumina TrueSeq DNA PCR-free kit and paired-end libraries were used to prepare libraries for the three strains. Library size and integrity were checked by running a DNA chip on an Agilent Technologies 2100 bioanalyzer. Passing criteria were DIN 7 and a library size between 450 and 800 bp. Then, sequencing was performed with a HiSeq 2500 instrument. Total reads produced were 2.96, 7.89, and 7.95 Gbp for P816, P822, and P824, respectively. Raw reads were processed and assembled in-house with Geneious 10.1.2 (3). Reads were trimmed to eliminate adapter and low-quality sequences with default parameters for the “trim ends” menu in Geneious. Reads shorter than 50 nucleotides (nt) or of low quality (*P* < 95% accuracy) were discarded. Filtered reads were assembled with Geneious and Spades v. 3.11.1 (4) using default parameters. Contigs were double filtered for adapter sequences with the same trim ends menu, and contigs less than 200 nt were discarded. In addition to the Illumina sequencing, BGI Genomics Co., Ltd. sequenced P824 with a PacBio RS II instrument. An aliquot of the same DNA extraction used for Illumina sequencing was used to prepare the library for SMRTbell sequencing. First, DNA was sheared with a Covaris g-TUBE, and then a DNA template prep kit 3.0, DNA/polymerase binding kit, and DNA sequencing reagent 4.0 were used according to the manufacturer’s protocols. P1 of 66% was in the optimal range, quality was 0.85, and the mean length of reads was 8,500 bp, with a total output of 841 Mbp. PacBio and Illumina reads were assembled in contigs separately with Geneious as described before, and contigs were used together to assemble the P824 genome in two molecules with Geneious. Contigs were assembled, ordered, and numbered following closely related reference genomes (strain EP1, GenBank accession numbers NZ_CP015115 and NZ_CP015116; and strain KACC10709, GenBank accession numbers NZ_CP016904 and NZ_CP016905). The assembled sequences at the end of each molecule overlapped, allowing the molecules to be circularized. A quality assessment of all genome sequences was done with QUAST version 5.0 (5), including determining genome size, number of contigs, and *N*_50_ and *L*_50_ values using default parameters (Table 1). Contigs were annotated with the Prokaryotic Genome Annotation Pipeline (PGAAP) of NCBI (6). The genome features are in the normal range of similar sequenced strains of R. solanacearum (Table 1).

**TABLE 1:** Assembly statistics and general feature summary of R. solanacearum strain genomes

Isolate	Genome size (Mbp)	No. of contigs	G+C content (%)	*N*_50_ value (kbp)	*L*_50_	No. of CDSs^*a*^	No. of pseudogenes	No. of tRNAs
P816	5.173	142	67.04	112	12	3,641	256	41
P822	5.374	181	66.54	94.6	17	4,564	287	50
P824	6.019	2	67.23			4,708	216	59

a CDSs, coding DNA sequences.

These genome sequences will contribute to the identification of genetic determinants of host pathogenicity in blueberry.

### Data availability.

These whole-genome shotgun projects have been deposited in DDBJ/EMBL/GenBank under the accession numbers MTBB00000000 (P816), PQWP00000000 (P822), CP025741 (P824 chromosome), and CP025742 (P824 megaplasmid). The versions described in this paper are the first versions, MTBB01000000, PQWP01000000, CP025741.1, and CP025742.1, respectively. Raw sequence data for each strain were deposited under SRA accession numbers SRR8292481 (P816), SRR8294950 (P822), SRR8295615 (P824, PacBio), and SRR8295614 (P824, Illumina).

## References

[1] Norman DJ, Bocsanczy AM, Harmon P, Harmon CL, Khan A 2018 First report of bacterial wilt disease caused by *Ralstonia solanacearum* on blueberries (*Vaccinium corymbosum*) in Florida. Plant Dis 102:438–438. doi:10.1094/PDIS-06-17-0889-PDN.

[2] Denny TP, Hayward AC 2001 Gram-negative bacteria: Ralstonia, p 151–174. *In* Schaad NW, Jones JB, Chun W (ed), Laboratory guide for identification of plant pathogenic bacteria, 3rd ed APS Press, St. Paul, MN.

[3] Kearse M, Moir R, Wilson A, Stones-Havas S, Cheung M, Sturrock S, Buxton S, Cooper A, Markowitz S, Duran C, Thierer T, Ashton B, Meintjes P, Drummond A 2012 Geneious Basic: an integrated and extendable desktop software platform for the organization and analysis of sequence data. Bioinformatics 28:1647–1649. doi:10.1093/bioinformatics/bts199. 22543367 PMC3371832

[4] Bankevich A, Nurk S, Antipov D, Gurevich AA, Dvorkin M, Kulikov AS, Lesin VM, Nikolenko SI, Pham S, Prjibelski AD, Pyshkin AV, Sirotkin AV, Vyahhi N, Tesler G, Alekseyev MA, Pevzner PA 2012 SPAdes: a new genome assembly algorithm and its applications to single-cell sequencing. J Comput Biol 19:455–477. doi:10.1089/cmb.2012.0021. 22506599 PMC3342519

[5] Gurevich A, Saveliev V, Vyahhi N, Tesler G 2013 QUAST: quality assessment tool for genome assemblies. Bioinformatics 29:1072–1075. doi:10.1093/bioinformatics/btt086. 23422339 PMC3624806

[6] Tatusova T, DiCuccio M, Badretdin A, Chetvernin V, Nawrocki EP, Zaslavsky L, Lomsadze A, Pruitt KD, Borodovsky M, Ostell J 2016 NCBI Prokaryotic Genome Annotation Pipeline. Nucleic Acids Res 44:6614–6624. doi:10.1093/nar/gkw569. 27342282 PMC5001611

